# Mutant prevention concentrations and phenotypic and genomic profiling of first-step resistance mechanisms to classical and novel β-lactams in *Pseudomonas aeruginosa*

**DOI:** 10.1128/aac.01942-24

**Published:** 2025-03-11

**Authors:** Miquel Àngel Sastre-Femenia, Maria Antonia Gomis-Font, Antonio Oliver

**Affiliations:** 1 Department of Microbiology, Hospital Universitari Son Espases, Instituto de Investigación Sanitaria Illes Balears (IdISBa), CIBERINFEC 219656 , Palma, Spain; Universita degli studi di roma La Sapienza, Rome, Italy

**Keywords:** *Pseudomonas aeruginosa*, mutant prevention concentration, beta-lactams, resistance, genomics

## Abstract

A growing number of novel antipseudomonal β-lactams have been introduced in recent years, but the emergence of resistance is still a major concern in the treatment of *Pseudomonas aeruginosa* infections. Here, we compared the mutant prevention concentrations (MPCs) and the nature of first-step resistant mutants to classical and novel β-lactams in *P. aeruginosa*. MPCs were determined in duplicate experiments for ceftazidime, ceftazidime/avibactam, ceftolozane/tazobactam, imipenem, imipenem/relebactam, meropenem, meropenem/vaborbactam, aztreonam, aztreonam/avibactam, and cefiderocol in PAO1, PAOMS (Δ*mutS*), and three extensively drug-resistant (XDR) clinical strains belonging to high-risk clones ST111, ST175, and ST235. Four mutants per strain and antibiotic, obtained from the highest concentration showing growth, were characterized through the determination of the susceptibility profiles and whole genome sequencing. Imipenem/relebactam presented the lowest MPC values, followed by ceftolozane/tazobactam. Overall, the MICs of the mutants were consistent with the antibiotic selection concentration, except for cefiderocol, which were much lower. MPCs were lower for ceftazidime/avibactam and imipenem/relebactam than those of the corresponding β-lactam alone. In contrast, MPCs of meropenem ± vaborbactam and aztreonam ± avibactam were identical in most strains. Ceftolozane/tazobactam and ceftazidime/avibactam derivatives presented mutations in *ampC*, *galU*, *cpxRS,* and/or in *bla_OXA-2_* when present in the parent strain (ST235). Cefiderocol mutants were mainly defective in iron-uptake systems, particularly PiuA/DC. All carbapenems had oprD as the first-step mechanism. Imipenem/relebactam, meropenem ± vaborbactam, and aztreonam ± avibactam selected mutations frequently included efflux pumps and regulators. Imipenem ± relebactam also selected *aroB* mutations. This work first describes the MPCs and first-step resistance mechanisms for classical and novel β-lactams in *P. aeruginosa*. The identified shared and differential resistance development patterns between the available classical and novel β-lactams should be helpful to guide treatment strategies for XDR *P. aeruginosa* infections.

## INTRODUCTION

*Pseudomonas aeruginosa*, a ubiquitous opportunistic pathogen considered one of the paradigms of antimicrobial resistance, is among the main causes of hospital-acquired and chronic infections associated with high morbidity and mortality (1, 2). Indeed*, P. aeruginosa* infections are estimated to be associated with over 300,000 annual deaths (3) and are classified as high priority in the 2024 WHO list for the need for research and development of new antibiotics (4). This growing threat results from the extraordinary capacity of this pathogen for developing resistance through chromosomal mutations and from the increasing prevalence of transferable resistance determinants (1, 5). *P. aeruginosa* has a non-clonal epidemic population structure, composed of a limited number of widespread clones, which are selected from a background of a large quantity of rare and unrelated genotypes that are recombining at high frequency (6). Indeed, several surveys have provided evidence of the existence of XDR global clones, denominated high-risk clones, disseminated in hospitals worldwide; ST235, ST111, and ST175 are among those most widespread (6, 7). Epidemic clones are also occasionally documented in chronic infections, but the main resistance threat in this setting is the frequent emergence of mutator (hypermutable) variants that catalyze the evolution of antimicrobial resistance (2).

The recent introduction of multiple novel β-lactam/β-lactamase inhibitor combinations including ceftolozane/tazobactam, ceftazidime/avibactam, imipenem/relebactam, meropenem/vaborbactam and aztreonam/avibactam, and the siderophore cephalosporin cefiderocol may help to mitigate, to some extent, the problem of XDR *P. aeruginosa* (8). However, despite most of these novel antibiotics being quite stable against classical β-lactam resistance mechanisms (such as the overexpression of the β-lactamase AmpC or efflux pumps), they are not fully exempt from resistance development, evidenced right upon their introduction into clinical practice (9, 10).

Indeed, emerging resistance mechanisms to these novel agents were already predicted by previous *in vitro* evolution experiments and frequently included gain-of-function mutations leading to the modification of the catalytic centers of the intrinsic AmpC or acquired OXA-2/OXA-10 β-lactamases (11–13), as well as mutations in iron transport systems in the case of cefiderocol (14). The use of mutator strains and XDR high-risk clone isolates in these evolution experiments was crucial for deciphering those resistance mechanisms, particularly when it required two or more simultaneous mutations or the presence of pre-existent first-step resistance mutations or horizontally acquired resistance determinants (11–15). Some recent works have also compared resistance development to some of the classical and novel β-lactams through serial *in vitro* passing of several clinical strains (16).

However, there is still a current lack of a systematic comparative analysis of the different classical and novel antipseudomonal β-lactams in terms of the genetic resistance barrier, the underlying genomic mechanisms, and the extent of cross-resistance or collateral susceptibility between these novel agents. Thus, in order to fill this gap, in this work, we performed resistant populations analysis, determined the mutant prevention concentrations (MPCs), and characterized at phenotypic and genomic levels the first-step resistance mechanisms to ceftazidime, ceftazidime/avibactam, ceftolozane/tazobactam, imipenem, imipenem/relebactam, meropenem, meropenem/vaborbactam, aztreonam, aztreonam/avibactam, and cefiderocol in wildtype, mutator, and XDR high-risk clones of *P. aeruginosa*.

## MATERIALS AND METHODS

### Strains, resistant population analysis, and MPCs

Wild-type reference strain PAO1 and its hypermutable *mutS*-deficient derivative PAOMS were used (17). Additionally, three previously characterized XDR clinical strains belonging to the high-risk clones ST111 [isolate NAV01-012: *oprD* (nt_991_InsAC), *dacB* (nt_664_InsGGCCT), *bla*_CARB-2_], ST175 [isolate 101-E5; *oprD* (Q142*), *ampR* (G154R), *mexZ* (G195D)], and ST235 [isolate 109-F7: *oprD* (nt_1205_InsC), *mexZ* (V48A), *bla*_OXA-2_] were used (13). The three isolates were determined to be resistant to all classical antipseudomonal β-lactams tested (ceftazidime, cefepime, piperacillin/tazobactam, imipenem, and meropenem). Moreover, they have been previously used for *in vitro* evolution experiments of ceftolozane/tazobactam, imipenem/relebactam, and cefiderocol resistance (13, 14). 1 mL of overnight cultures, containing approx. 10⁹ CFU/mL, and serial dilutions were seeded in Mueller–Hinton (MH) agar plates containing a range of different concentrations (0–32 µg/mL) of ceftazidime, ceftazidime + 4 µg/mL avibactam, ceftolozane + 4 µg/mL tazobactam, imipenem, imipenem + 4 µg/mL relebactam, meropenem, meropenem + 8 µg/mL vaborbactam, aztreonam, aztreonam + 4 µg/mL avibactam or cefiderocol for each strain in duplicate independent experiments. To avoid the inoculum effect, 200 µL was plated in each of five plates for direct cultures. CFUs were enumerated and plotted after 24 h of incubation at 37°C. MPC was defined as the lowest concentration of antibiotic yielding no growth in any of the seeded plates for a given strain and antibiotic (18, 19). Two mutants per duplicated experiment, antibiotic, and strain tested, obtained from the plates containing the highest antibiotic concentration showing growth were passed in antibiotic-free media and stored frozen at −80°C until characterization through the determination of the susceptibility profiles and whole-genome sequencing as described below. Thus, a total of 200 mutants were characterized (2 mutants × 2 experiments × 5 strains × 10 antibiotics).

### Susceptibility testing

MICs of ceftazidime, ceftazidime/avibactam, ceftolozane/tazobactam, imipenem, imipenem/relebactam, meropenem, meropenem/vaborbactam, aztreonam, and aztreonam/avibactam (0.12–128 mg/L) were determined by broth microdilution in cation-adjusted MH (CAMHB). MICs of cefiderocol (0.015–128 mg/L) were determined using iron-depleted cation-adjusted MH (IDCAMHB) broth following EUCAST guidelines. EUCAST 2024 clinical breakpoints were used for interpretation (https://www.eucast.org). Additionally, MICs of cefiderocol were determined, for comparative purposes, in conventional CAMHB. Moreover, the phenotypic characterization of cefiderocol mutants was completed by disk diffusion susceptibility testing according to EUCAST guidelines.

### Whole-genome sequencing

Previously defined and validated protocols were used with slight modifications (14). Total DNA was isolated using a commercial capture system (High Pure PCR Template Preparation Kit, Roche Diagnostics), and indexed paired-end libraries were generated using the Illumina DNA Prep library preparation kit (Illumina Inc., USA) and then sequenced on an Illumina NovaSeq 6000 platform. The reads for each isolate were mapped against the genome of the *P. aeruginosa* reference strain PAO1 (RefSeq accession number NC_002516.2) using Bowtie 2 software, version 2.2.6 (http://bowtie-bio.sourceforge.net/bowtie2/index.shtml) (20). Pileups and raw files of the mapped reads were obtained using SAMtools, version 0.1.16 (https://sourceforge.net/projects/samtools/files/samtools/) (21) and Picard, version 1.140 (https://github.com/broadinstitute/picard). Read alignments surrounding all putative indels were realigned using the Genome Analysis Toolkit, version 3.4-46 (https://www.broadinstitute.org/gatk/) (22). The list of SNPs was compiled from the raw files that met the following criteria: a quality score of >50, a root mean square (RMS) mapping quality of >25 and a coverage depth of >30. Microindels were extracted from the total pileup files using the following criteria: a quality score of >250, an RMS mapping quality of >25, and a coverage depth of >30. SNPs and indels for each isolate were annotated using SnpEff software version 4.3 (http://snpeff.sourceforge.net/index.html) (23). Finally, large chromosomal deletions were analyzed with SeqMonk version 1.47.2 (https://www.bioinformatics.babraham.ac.uk/projects/seqmonk/) and R v4.2.3 within the RStudio v0.99.896 platform (24). Acquired mutations in OXA-2 for ST235 were detected from *de novo* assembled reads obtained using SPAdes v3.15 with default options.

## RESULTS

### Resistant populations analysis and MPCs for classical and novel antipseudomonal β-lactams

**Fig 1:**
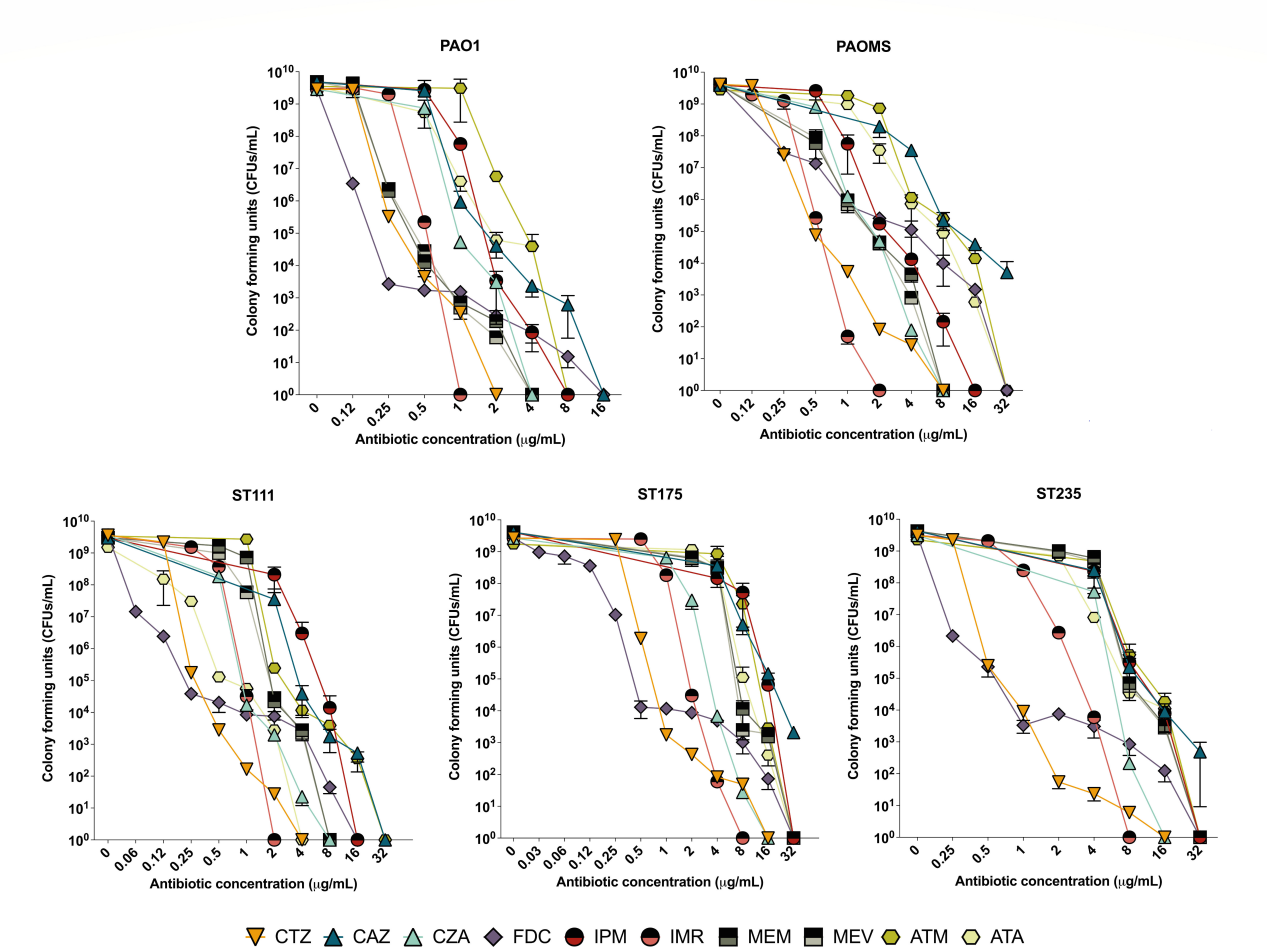
Resistant populations analysis of the tested classical and newer β-lactam for wild-type strain PAO1, its mutator derivative PAOMS, and the three high-risk clone isolates.

Results from resistant populations analysis are shown in Fig. 1, whereas Table 1 collects MICs and MPCs data for all strains and antibiotics tested. As shown, MPCs were lowest for imipenem/relebactam, followed by ceftolozane/tazobactam in all strains tested, including wild-type PAO1, its mutator derivative, and the three XDR high-risk clone isolates. MPCs were lower for ceftazidime/avibactam and imipenem/relebactam than those of the corresponding β-lactam without β-lactamase inhibitor. In sharp contrast, MPCs of meropenem and meropenem/vaborbactam were identical in the five strains tested. On the other hand, MPCs of aztreonam/avibactam were equal in four strains (and lower in one) to those of aztreonam. Finally, cefiderocol MPCs were high for all strains, and the highest MPC to MIC ratios were obtained for this antibiotic. Moreover, as shown in Fig. 1, cefiderocol decreased CFU counts at low concentrations consistently with low MICs but failed to fully suppress growth in a large concentration range, determining its high MPC values.

**TABLE 1:** MICs and MPCs of classical and newer β-lactams for the panel of strains tested^*a*^

Atb	PAO1		PAOMS		ST111		ST175		ST235	
	MIC	MPC	MIC	MPC	MIC	MPC	MIC	MPC	MIC	MPC
CTZ	0.25	2	0.25	8	0.5	4	1	16	1	16
CAZ	1	16	8	>32	8–16	32	16	>32	16	>32
CZA	1	4	1	8	1	8	2	16	2	16
FDC	0.06	8–16	0.25	32	0.03	16	0.015	32	0.5–1	32
IPM	1	8	2	16	8	16	16	32	16	32
IMR	0.25	1	0.25	2	0.5	2	1–2	8	1–2	8
MEM	0.25	4	2	8	4	8	8	32	8	32
MEV	0.25	4	2	8	4	8	8	32	8	32
ATM	2	8	8	32	4	32	8	32	8	32
ATA	2	8	8	32	1	4	8	32	8	32

^*a*^ Atb, antibiotic; CTZ, ceftolozane/tazobactam; CAZ, ceftazidime; CZA, ceftazidime/avibactam; FDC, cefiderocol; IPM, imipenem; IMR, imipenem/relebactam; MEM, meropenem; MEV, meropenem/vaborbactam; ATM, aztreonam; ATA, aztreonam/avibactam.

### Phenotypic profiling and first-step resistance mechanisms genomics for classical and novel antipseudomonal β-lactams

**Fig 2:**
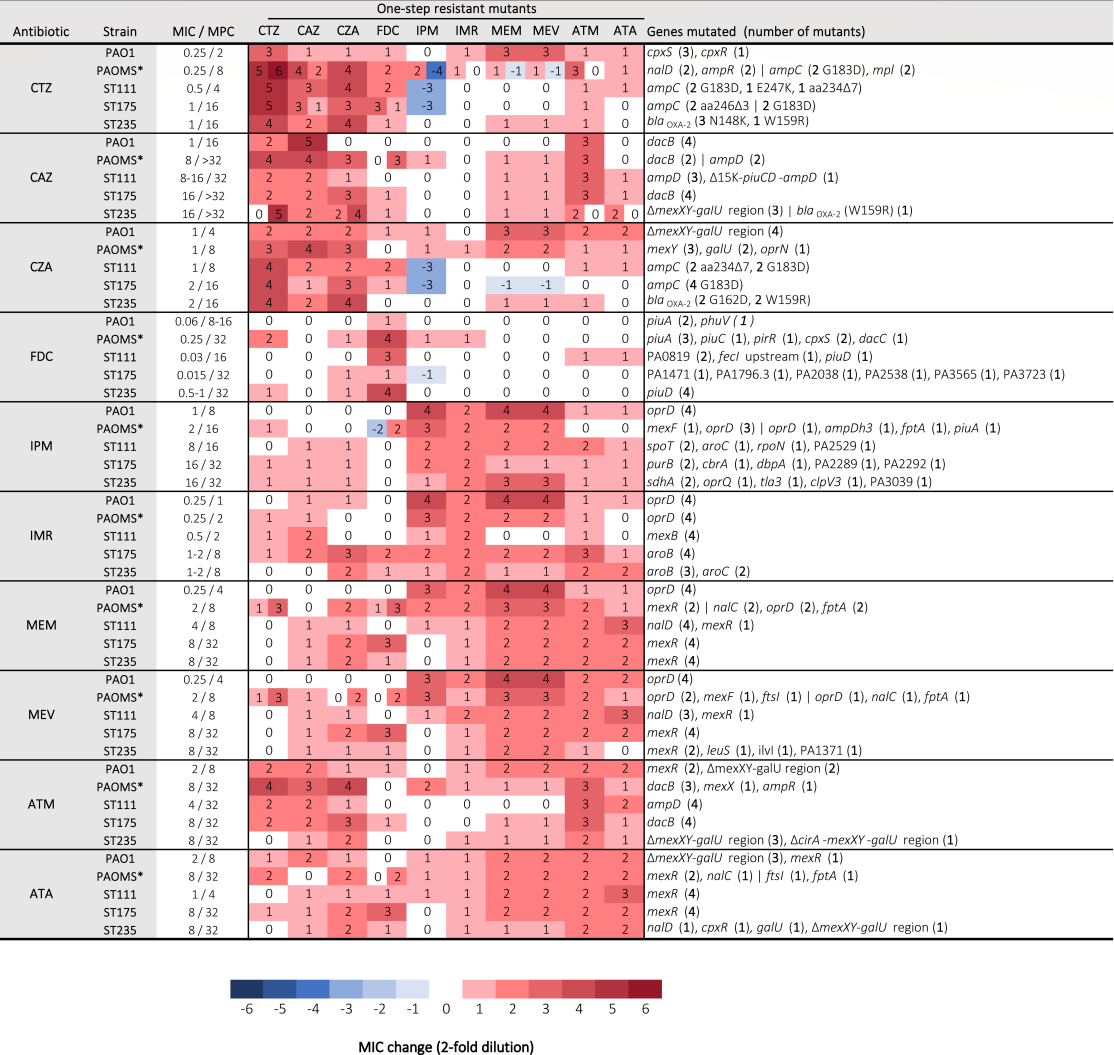
Phenotypic and genomic characterization of the four resistant mutants studied per strain and antibiotic. Median MIC changes are indicated when all were within 1 twofold dilution. When variation was over 1 twofold dilution, varying MICs are shown independently. *For the PAOMS strain, 15–38 mutations were detected for each mutant, but only those related to β-lactam resistance are shown.

The susceptibility profiles and whole-genome sequence resistome analysis for the 200 first-step resistant mutants analyzed for the different strains and antibiotics are summarized in Fig. 2 and fully listed in Table S1. As could be expected, the vast majority of the resistant mutants showed increased MICs for the selecting agents, with values in the range expected by the concentrations of antibiotics in the plates from which they were isolated. Notable exceptions were cefiderocol-resistant mutants, since they systematically showed much lower MIC values than the concentrations on the plates from which they were isolated. In other words, for cefiderocol, in contrast to all other β-lactams tested, a strong discordance between MPC values and MIC of first-step resistant mutants was evidenced (see below).

Cross-resistance was high between ceftolozane/tazobactam, ceftazidime and ceftazidime/avibactam, as well as for imipenem, imipenem/relebactam, meropenem, and meropenem/vaborbactam. It was also high for aztreonam and aztreonam/avibactam and to some extent also with meropenem and meropenem/vaborbactam. Overall, cefiderocol showed the lowest cross-resistance with other agents, whereas imipenem showed collateral susceptibility in several ceftolozane/tazobactam and ceftazidime/avibactam-resistant mutants.

Regarding resistance mechanisms, most (>95%) of the mutants, except those derived from the *mutS* strain, showed single resistance mutations, with only a very few showing two (or even three occasionally) mutations, consistent with expected results from MPC assays (one-step resistant mutants). Ceftazidime resistance was most frequently driven by mutation in *ampD* or *dacB* (PBP4) *ampC* regulators. Ceftolozane/tazobactam and ceftazidime/avibactam resistance mechanisms included AmpC Ω-loop mutations as well as mutations in the catalytic center leading to previously described extended spectrum variants in the OXA-2 producing strain. Particularly noteworthy, the four PAO1 ceftolozane/tazobactam mutants studied showed mutations in the CpxRS two-component regulator and showed cross-resistance, particularly with meropenem (and meropenem/vaborbactam). Some ceftazidime and ceftazidime/avibactam mutants showed large genomic deletions in the *galU* region. Cefiderocol-resistant mutants mostly showed mutations in iron transport systems, particularly frequently in PiuA/D-C, explaining low cross-resistance with other β-lactams. All carbapenems (imipenem, imipenem/relebactam, meropenem and meropenem/vaborbactam) had *oprD* as the first primary mutational target. When *oprD* was already inactivated in the parent strain, mutations in MexAB-OprM regulators (*mexR*, *nalC*, or *nalD*) were frequently selected by imipenem/relebactam (but not imipenem alone), meropenem and meropenem/vaborbactam. Indeed, mutational resistance patterns were nearly identical for meropenem and meropenem/vaborbactam. Imipenem/relebactam-resistant mutants frequently showed mutations in *aroB* or *aroC* genes, whereas mutations selected by imipenem alone were more variable. Finally, aztreonam and aztreonam/avibactam frequently selected mutations in MexAB-OprM regulators and large genomic deletions in the *galU* region, whereas aztreonam alone additionally selected mutations leading to AmpC overexpression.

### Comparative analysis of susceptibility testing methods and resistome analysis for cefiderocol mutants

In order to dissect the discordance between MPC values and MIC of first-step cefiderocol-resistant mutants, an extended comparative analysis of susceptibility testing methods and resistome analysis of the mutants obtained in MH agar plates containing cefiderocol was performed. For this purpose, IDCAMHB MICs were compared with those obtained in conventional broth microdilution and by the EUCAST disk diffusion method. As shown in Table 2, MICs obtained by conventional broth microdilution correlated well with the concentration of cefiderocol in the MH agar plates from which they were selected. Moreover, disk diffusion results evidenced decreased cefiderocol susceptibility in all mutants, even reaching in most cases clinical resistance according to EUCAST breakpoints.

**TABLE 2:** Comparative analysis of susceptibility testing methods and resistome analysis of the mutants obtained in Mueller–Hinton agar plates containing cefiderocol

Strain	Experiment	Cefiderocol concentration in plates (µg/mL)	Mutant	MIC CA-MH^*a*^	MIC IDCA-MH^*a*^ (*R* > 2)	Zone diameter (mm)^*b*^ (*R* < 22)	Acquired mutations^*c*^
PAO1			WT	0.5	0.06	26	
	1	8	1	8	0.12	20	PA2435 (A606V), *opmK* (A203T)
			2	8	0.12	20	*hutC* (Y181X), *piuA* (aa30Δ2)
	2	4	1	16	0.12	19	*piuA* (aa665Δ2)
			2	8	0.25	19	*phuV* (V114D), *pilB* (N349K)
PAOMS			WT	1	0.25	26	
	1	16	1	32	4	14	*dacC* (E298G), *piuA* (Q685X)
			2	32	4	13	*cpxS* (V235A), *dacC* (E298G), *piuA* (Q685X)
	2	16	1	16	4	17	*piuA* (Q548X)
			2	16	4	17	*pirR* (nt387Δ1), *piuC* (W223X)
ST111			WT	0.12	0.03	30	
	1	8	1	8	0.12	23	PA0819 (T77I)
			2	8	0.25	22	PA0819 (T77I)
	2	8	1	16	0.25	23	Upstream_*fecI* (nt4367239InsA)
			2	16	0.25	22	*piuD* (E157X), *ynbD* (R261H)
ST175			WT	0.5	0.015	28	
	1	16	1	16	0.03	18	upstream_PA3565 (3996534InsAGTTAA)
			2	16	0.03	19	PA1471 (aa48InsT), upstream_PA2038 (2229098InsAGA)
	2	16	1	32	0.03	19	PA1796.3 (aa25InsE), PA2538 (aa20InsSC)
			2	32	0.03	19	upstream_PA3723 (4168847ΔT)
ST235			WT	1–2	0.5–1	26	
	1	16	1	32	8	16	*piuD* (G354V)
			2	32	8	16	*piuD* (Y720X), *oprN* (A234T)
	2	16	1	32	8	16	*piuD* (Q604X), PA2108 (A265T)
			2	32	8	16	*piuD* (nt1772Δ13)

^*a*^ Broth microdilution MIC results in cation-adjusted Muller–Hinton (CA-MH) and iron-depleted cation-adjusted Muller–Hinton (IDCA-MH). EUCAST breakpoints (IDCA-MH) indicated.

^*b*^ Disk diffusion zone diameters in Muller–Hinton agar plates. EUCAST resistance breakpoint indicated. EUCAST area of technical uncertainty (ATU) for cefiderocol is 20–21 mm.

^*c*^ For PAOMS derivatives, only mutations relevant for cefiderocol resistance are listed.

## DISCUSSION

XDR, and particularly carbapenem-resistant, *P. aeruginosa* was included in the top (critical) priority in the first (2018) WHO list for the need for research and development of new antibiotics (25) and is still considered a high priority in the 2024 updated document (4). The introduction over the last decade of multiple novel β-lactam/β-lactamase inhibitor combinations including ceftolozane/tazobactam, ceftazidime/avibactam, imipenem/relebactam, meropenem/vaborbactam and aztreonam/avibactam, and the siderophore cephalosporin cefiderocol may help to mitigate, to some extent, the problem of antibiotic-resistant *P. aeruginosa*. Indeed, the growing diversity of therapeutic options is also accompanied by a growing diversity and complexity of resistance mechanisms, determining the need for establishing personalized precision-medicine treatment strategies, taking into consideration not only the patient and infection characteristics but also the specific pathogen, strain, and resistance mechanisms produced (26). While some of these agents have a unique target not covered by most others, such as cefiderocol (and to some extent aztreonam/avibactam) for MBL-producing strains or ceftazidime/avibactam for class A carbapenemases, a major piece of the puzzle is how to position these novel β-lactams in infections by strains showing resistance mechanisms conferring resistance to classical β-lactams. One of the variables (not the only one) to be considered for such purpose is the evaluation of the stability against resistance development. The analysis of the presence of spontaneous resistant subpopulations for a range of antibiotic concentrations and especially the determination of the MPCs is one of the classical approaches for such purposes (18, 27). Here, we analyzed these parameters for the first time in all currently commercialized novel antipseudomonal β-lactams β-lactamase inhibitor combinations (and the corresponding β-lactams alone) and cefiderocol. MPCs were lowest for imipenem/relebactam followed by ceftolozane/tazobactam in all strains tested. MPCs were lower for the combination than for the β-lactam alone in some cases (ceftazidime/avibactam and imipenem/relebactam) but not in others (meropenem/vaborbactam and aztreonam/avibactam). On the other hand, cefiderocol MPCs were high for all strains, and the highest MPC to MIC ratios were obtained for this antibiotic. Remarkably, cross-resistance development varied significantly across agents. Cross-resistance was high between ceftolozane/tazobactam, ceftazidime and ceftazidime/avibactam, as well as for imipenem, imipenem/relebactam, meropenem, and meropenem/vaborbactam. Cross-resistance was also high for aztreonam and aztreonam/avibactam and to some extent also with meropenem and meropenem/vaborbactam. Overall, cefiderocol showed the lowest cross-resistance with other agents, whereas imipenem showed collateral susceptibility in several ceftolozane/tazobactam and ceftazidime/avibactam resistant mutants.

Regarding the involved resistance mechanisms, mutations in the catalytic center of AmpC were selected by ceftolozane/tazobactam and ceftazidime/avibactam in the two XDR clinical strains hyperproducing AmpC, as well as in the *mutS* strain, simultaneously with mutations leading to *ampC* overexpression. Thus, these results are consistent with previous *in vitro* and *in vivo* works showing that resistance development to these agents requires the simultaneous combination of AmpC overexpression and structural modification (9, 11, 28). On the other hand, ceftolozane/tazobactam and ceftazidime/avibactam primarily selected extended-spectrum OXA-2 mutations in the strain producing this acquired β-lactamase. These results were thus in agreement with information with clinical strains and alerts on the need to consider the presence of narrow-spectrum OXA such as OXA-2 and OXA-10 when designing personalized treatments (9, 29). Moreover, these mutations were also selected by ceftazidime alone, as previously documented *in vivo,* alerting of the potential cross-resistance development between classical and novel β-lactams (30).

Mutations (*mexR*, *nalB*, or *nalC*) leading to the overexpression or structural modification (*mexB*) of the efflux pump MexAB-OprM were the most broadly selected mutations, including meropenem, meropenem/vaborbactam, imipenem/relebactam, aztreonam and aztreonam/avibactam. Among *mexB* mutations, the R620C substitution, selected by imipenem/relebactam, is particularly noteworthy. Indeed, this substitution is located in the second periplasmic loop and situated in the lining of the pump’s ligand binding pockets (31). Remarkably, this same mutation has been previously noted to be selected by imipenem/relebactam exposure in *in vitro* evolution experiments (12, 13) and treated patients (10) and is therefore speculated to be involved in the recognition and extrusion of relebactam by MexAB-OprM. Moreover, different mutations in the same region have been associated with the acquisition of resistance to the combination of cefepime with zidebactam (31, 32). Other noteworthy resistance mutations include those on the CpxRS envelope stress two-component regulator, particularly selected in PAO1 by ceftolozane/tazobactam and showing cross-resistance with meropenem (and meropenem/vaborbactam). CpxRS has been shown to be involved in cellular hysteresis response to β-lactam antibiotics (33) and the regulation of the expression of MexAXB-OprM (34). However, it has been also associated with the acquisition of ceftolozane/tazobactam resistance in *in vitro* assays in strains defective in the efflux pump by mechanisms that still need to be elucidated (35).

Other frequent mutations, selected specifically by imipenem or imipenem/relebactam, were those on the components of the shikimate pathway (aromatic amino acid metabolism) *aroB* (3-dehydroquinate synthase) or *aroC* (chorismate synthase) (36). While the specific role of such mutations in imipenem (and imipenem/relebactam) resistance needs to be further investigated, selection of such mutations by this antibiotic was previously noted as well in *in vitro* evolution experiments (12, 13). Moreover, the analysis of transposon mutant collections has also evidenced, at least in the case of *aroB* (*aroC* mutants are not available), their effect on β-lactam resistance (37, 38). Finally, mutations in iron transport systems were selected in most cefiderocol mutants as first step, even if MICs remained quite low in most cases. Noteworthy was the selection by ceftazidime of a large deletion including *ampD* (*ampC* regulator) and *piuAC* regions, as recently evidenced in a clinical isolate (39). In any case, a major issue evidenced is the high discordance between the cefiderocol MPC values obtained in MHA plates and the subsequent MIC of the mutants in IDCAMHB. In contrast, MICs obtained by conventional broth microdilution correlated well with the concentration of cefiderocol in the MHA plates from which they were selected. Moreover, disk diffusion results evidenced decreased cefiderocol susceptibility in all mutants, even reaching in most cases clinical resistance according to EUCAST breakpoints. Thus, while MHA is considered appropriate for cefiderocol susceptibility testing, such as in reference disk diffusion method, our work evidences that mutants selected in MHA do not necessarily show equally increased MICs when tested in IDCAMHB. Indeed, cefiderocol susceptibility testing remains a challenge for clinical microbiology laboratories, with major discrepancies between different methods (40).

In summary, through the analysis of resistant populations, the determination of the MPCs and the characterization of first-step resistant mutants, we have identified both shared and differential phenotypic and genomic resistance traits between the different classical and novel β-lactams, as well as between the different *P. aeruginosa* genotypes tested. Thus, the provided information should be helpful to guide the establishment of treatment strategies for XDR *P. aeruginosa*. Subsequent initiatives should keep incorporating novel β-lactams currently under clinical development such as cefepime/zidebactam and cefepime/taniborbactam.

## Data Availability

The complete sequences of the 200 mutants studied have been deposited in the European Nucleotide Archive under project accession PRJEB78485.

## SUPPLEMENTAL MATERIAL

Table S1 aac.01942-24-s0001.xlsx Phenotypic and genomic characterization of the studied mutants.

ASM does not own the copyrights to Supplemental Material that may be linked to, or accessed through, an article. The authors have granted ASM a non-exclusive, world-wide license to publish the Supplemental Material files. Please contact the corresponding author directly for reuse.
